# Development of Intelligent Composite Materials from Polyvinyl Alcohol (PVA) and *Clitoria ternatea* L. Anthocyanin Extract for Shrimp Freshness Monitoring

**DOI:** 10.3390/polym18060684

**Published:** 2026-03-11

**Authors:** Diana Carmona-Cantillo, Gustavo Gonzalez-Muñoz, Alexis López-Padilla, Fabian Rico-Rodríguez, Rodrigo Ortega-Toro

**Affiliations:** 1 Food Packaging and Shelf-Life Research Group (FP&SL), Food Engineering Department, Universidad de Cartagena, Avenida del Consulado Calle 30 No. 48–152, Cartagena de Indias 130015, Colombia; dcarmonac1@unicartagena.edu.co; 2 Research Group on Applied Transformation of Industrial and Agro-Industrial Matrices (ITMIA), Food Engineering Department, Universidad de Cartagena, Avenida del Consulado Calle 30 No. 48–152, Cartagena de Indias 130015, Colombia; ggonzalezm2@unicartagena.edu.co (G.G.-M.); alopezp2@unicartagena.edu.co (A.L.-P.); fricor@unicartagena.edu.co (F.R.-R.)

**Keywords:** anthocyanin, pH-sensitive films, color indicator, biofilm, shrimp, *Clitoria ternatea* L.

## Abstract

The development of bioplastic films represents an alternative to conventional plastics and an opportunity for applications in intelligent packaging. The present study aimed to develop a smart material based on poly (vinyl alcohol) (PVA) incorporated with *Clitoria ternatea* L. extract, capable of monitoring shrimp freshness through colour changes associated with pH variations. The films were prepared using the casting method and characterised in terms of their physical, mechanical, structural, and functional properties. The incorporation of the anthocyanin extract (EAC) significantly intensified the colouration of the films, decreasing lightness (L*) from 88.7 to 37.1 and modifying the chromatic parameters (b from −0.16 to −22.34). Thickness increased from 109.5 μm to 184 μm as the extract concentration was raised, while water vapour permeability ranged from 0.77 to 1.79 g·m/m^2^·s·Pa, evidencing modifications in the structure of the polymeric matrix. From a mechanical standpoint, tensile strength decreased from 26.0 MPa to 15.2 MPa, and the elastic modulus was reduced by approximately 75.0 MPa, whereas the percentage elongation at break increased from 75.2% to 92.4%, confirming the plasticising effect of the extract. Functionally, the films exhibited a visible transition from blue to green during the refrigerated storage of shrimp, corresponding to increases in pH from 6.6 to 9.2 and total volatile basic nitrogen (TVB-N) values from 3.92 to 67.7 mg N/100 g. The formation of TVB-N followed first-order kinetics (R^2^ = 0.997), confirming the sensitivity of the system as a freshness indicator. These results demonstrate the potential of PVA–anthocyanin films as intelligent colorimetric sensors for monitoring the freshness of protein-rich foods.

## 1. Introduction

The persistent resistance of petroleum-based plastics to degradation has created serious ecological challenges. These materials contaminate soil and water, introducing microplastics into the food chain and posing potential health risks to humans [1].

The development of bioplastic films represents a promising alternative to conventional plastics, potentially offering a reduced environmental impact due to the use of polymeric matrices and natural additives that may exhibit biodegradability under appropriate conditions. Simultaneously, increasing consumer awareness of food safety and quality has promoted the implementation of intelligent packaging systems capable of real-time monitoring of product conditions. These systems enable evaluation of key parameters such as quality, safety, microbial contamination, and freshness during storage and distribution. Among the most innovative technologies are colorimetric indicator films, which signal changes in food freshness or quality through visually detectable color variations, eliminating the need for additional physical or chemical analyses during preservation [2].

*Clitoria ternatea* L., commonly known as butterfly pea flower, has garnered growing interest due to its natural pH-dependent color-changing ability, attributed to its anthocyanins. This sensitivity has been applied in food, traditional medicine, and biotechnology [3]. Beyond conventional uses, rising demand for clean-label products has driven valorization of their extracts in global markets. Recent research focuses on enhancing anthocyanin content through cultivation techniques, selective breeding, or bio-stimulation methods like elicitors and plasma technology, potentially increasing commercial value and nutritional/therapeutic properties, positioning butterfly pea as a sustainable resource for food, cosmetic, and pharmaceutical industries [4].

Currently, *C. ternatea* L. anthocyanins particularly ternatins, a subclass responsible for intense pigmentation and strong antioxidant properties are under investigation for intelligent material development. These natural pigments serve as eco-friendly, healthy alternatives to synthetic dyes and function as bioactive ingredients in nutraceuticals and beverages due to anti-inflammatory, antidiabetic, and neuroprotective effects [5].

Promising approaches for intelligent materials include polymeric films incorporating pH-sensitive pigments, offering low-cost, high-efficacy indicators. In this context, combining poly (vinyl alcohol) (PVA) with *Clitoria ternatea* L. extracts holds significant potential for creating active, responsive films that exhibit chromatic changes in response to environmental shifts, such as pH variations or specific compound presence [6]. Intelligent packaging systems generally respond to changes in time, temperature, or pH, with pH indicators being the most common in the food industry due to high sensitivity. While both synthetic and natural colorants are used, natural pigments offer key advantages: non-toxicity, abundance, biodegradability, and additional functionalities [2,6].

Among natural options, *C. ternatea* L. anthocyanins stand out for their notable stability against light and heat, along with distinct color changes across acidic, neutral, and basic pH conditions, making them especially suitable for pH-indicator films. Compared to other anthocyanins, those from *Clitoria ternatea* L. show relatively good light and heat stability and clear differentiation in neutral, acidic, and basic environments. However, studies indicate that color changes in films incorporating these anthocyanins can be rapid, complicating detection of food spoilage under real conditions. Additionally, reversibility of color indication has not been consistently demonstrated, limiting efficiency. Thus, improving pH-responsive color reversibility is essential to enhance these films’ effectiveness as indicators [7,8]. In this framework, the present research aimed to develop an intelligent material based on poly (vinyl alcohol) (PVA) incorporated with *Clitoria ternatea* L. extract, capable of monitoring shrimp freshness through pH-associated color changes.

## 2. Materials and Methods

### 2.1. Materials

The blue pea flower (*Clitoria ternatea* L.) used in this study was purchased from Granitos de Paz (Cartagena, Colombia). This species is a cultivated ornamental species and is not listed as endangered under the IUCN Red List or CITES. The plant material was obtained from a commercial supplier, and no wild collection was involved. The shrimp used for application tests were supplied by OCÉANOS S.A., (Cartagena, Colombia). The reagents required for the extraction process, including absolute Ethanol (≥99.5%, analytical grade), hydrochloric acid (HCl; 37%, analytical grade), and sodium hydroxide (NaOH; ≥98%, pellets, analytical grade), were provided by Elementos Químicos Ldta (Bogotá, Colombia). Poly (vinyl alcohol) (PVA, Mw ≈ 85,000–124,000 g/mol, degree of hydrolysis 87–89%, purity ≥ 99%) was obtained from Sigma-Aldrich, (St. Louis, MO, USA).

### 2.2. Extraction Process of Butterfly Pea Flower Extract

To obtain the extract from Asian pigeonwings flowers (*Clitoria ternatea* L.), petals and sepals were separated, with petals used as the extraction material. They were dehydrated in a tray dryer (Gourmia Model# GFD1680) at 55 °C until constant weight was reached. The dried petals were then ground using a mill, and the resulting powder was stored in hermetic bags at −20 °C until use. The extraction followed the methodology of Chen et al. and Handayani et al. [9,10] with modifications, using an absolute ethanol/0.1 N HCl solution (85:15, *v*/*v*). The powdered flower sample was immersed in the solvent at a 1:20 (*w*/*v*) ratio, stirred at room temperature for 1 h in complete darkness (the container was wrapped in aluminum foil). After this period, the mixture was centrifuged (Z 206 A) at 4000 rpm for 10 min. The supernatant was collected and neutralized with 0.1 M NaOH to pH 6.0. It was then stored at 5 °C for 24 h to allow sugar sedimentation. Finally, the supernatant was separated using a micropipette and stored in a dark container at −20 °C until further use.

### 2.3. Characterization of Butterfly Pea Flower Extract (Clitoria ternatea *L*.)

#### 2.3.1. Maximum Wavelength (λmax) Measurement

The λ_max_ of the obtained extract was determined using a spectrophotometer BK-UV1900PC (BIOBASE Group, Jinan, China) over a wavelength range of 400–800 nm. A 1 mL aliquot of the extract was diluted to 10 mL with absolute ethanol in a volumetric flask. Absorbance was measured at 5 nm intervals within the specified range. Measurements were performed in quartz cuvettes with a 1 cm optical path length. Each sample was measured in triplicate (three independent dilutions prepared from the same extract), and the average spectrum was reported. Blanks (solvent without extract) were subtracted automatically.

#### 2.3.2. Total Anthocyanin Content Measurement

Following the method of Wrolstad et al. [11] with modifications, total anthocyanin content was calculated by diluting a concentrated aliquot of the flower extract with buffer solutions at pH 1.0 (0.025 M potassium chloride buffer) and pH 4.5 (0.4 M sodium acetate buffer). Absorbance was measured at 520 nm and 700 nm using a BIOBASE BK-UV1900PC spectrophotometer (BIOBASE Group, Jinan, China).

### 2.4. Film Preparation

Film-forming solutions were prepared according to the formulations presented in Table 1. A control film (pure PVA) was prepared by dissolving 4 g of PVA in 200 mL of distilled water (2% *w*/*v*) under magnetic stirring. Glycerol was added as a plasticizer at 25% (*w*/*w*) relative to the polymer mass, and the mixture was stirred for 15 min. The solution was degassed under vacuum, filtered, poured into Teflon trays, and dried at 40–45 °C for 9 h. For the extract-containing formulations (PA-A5, PA-A10, PA-A20, and PA-A30), the aqueous anthocyanin extract of *Clitoria ternatea* L. was incorporated by partially replacing distilled water at 5%, 10%, 20%, and 30% (*v*/*v*) of the total solvent volume (200 mL), respectively. The total solvent volume was kept constant at 200 mL in all formulations. The films were conditioned in desiccators containing saturated magnesium nitrate solution (53% RH) at 30 °C for 3–5 days prior to characterization.

### 2.5. Film Characterization

#### 2.5.1. Gloss

Gloss was measured at a 60° angle according to ASTM D523-89 [12] using a flat-surface gloss meter (3NH YG268 multi-angle gloss meter, Minolta, Langenhagen, Germany). A total of seven films were evaluated, with three measurements performed on each individual sample. Results were expressed in gloss units (GU).

#### 2.5.2. Color

Film color was measured using a portable colorimeter (CHN Spec CS-10), which provided CIE Lab* coordinates, hue angle (h), and chroma (c). Luminosity is represented on the vertical axis, while the horizontal axes indicate orientation toward red-green and yellow-blue colors [13]. Additionally, film color was monitored over six non-consecutive days to assess possible chromatic variations over time. Color differences relative to the reference sample were calculated by determining ΔL*, Δa*, and Δb* values. The total color difference (ΔE*) was then obtained using the corresponding Equation (1).

(1) $$∆E\;*=\sqrt{{∆L\;*}^{2}+{∆b\;*}^{2}+{∆a\;*}^{2}}$$

#### 2.5.3. Transmittance and Opacity

The methodology described in [14] was implemented with some modifications. Film transmittance was recorded in the UV-visible range using a spectrophotometer (BIOBASE BK-UV1900PC UPR19G0005, Biobase Biodustry (Shandong) Co., Ltd., Jinan, China). Films were cut into 1 × 3 cm strips and fixed to the inner wall of the cuvette for measurement. Opacity was determined using Equation (2).

(2) $$\mathrm{Opacity}=\frac{\mathrm{Absorbance}\;(600\;nm)}{\mathrm{Thickness}\;(mm)}$$

#### 2.5.4. Thickness

Film thickness was measured with a digital micrometer (TL268 TOP EU (Proster Trading Limited, Hong Kong, China). Seven random measurements were taken per film to report the mean value and standard deviation.

#### 2.5.5. Water Vapor Permeability (WVP)

WVP was determined gravimetrically following the procedure of Sri Agustini et al. [15] with several modifications. Environmental conditions were controlled to establish a humidity gradient between 52.8% RH and 100% RH at 25 °C. Defect-free films were selected for evaluation. Payne-type permeation cups filled with distilled water exposed one film surface to 100% RH. These cups were placed in cabinets maintained at 25 °C and 52.8% RH using saturated magnesium nitrate solutions. To enhance applicability for high-water-activity products, the free surface of the film during preparation was exposed to lower RH. Containers were weighed systematically on a high-precision analytical balance (sensitivity 0.0001 g). Once stable conditions were reached, water vapor transmission rate (WVTR) was calculated from the slope of the linear regression of weight loss over time and divided by the film area. The procedure was repeated three times, reporting mean values with standard deviation.

#### 2.5.6. Moisture Content and Water Solubility

The methodology proposed in [16] was used. Film samples (2 × 2 cm) were initially weighed (W_0_), then dried in an oven at 60 °C to constant weight (W_1_). Samples were immersed in distilled water at a 1:10 (film: water) ratio for 24 h. Finally, they were dried again to constant weight (W_2_). Moisture content and solubility were calculated using Equations (3) and (4).

(3) $$\%\mathrm{Moisture}=\frac{W0-W1}{W1}\times 100\%$$

(4) $$\%\mathrm{Solubility}=\frac{W1-W2}{W2}\times 100\%$$

#### 2.5.7. Water Absorption Capacity

Water absorption capacity was measured to determine the amount of water a sample can retain under controlled conditions [17]. Samples were placed in a desiccator with calcium chloride (0% RH). Weights were recorded every 24 h until constant dry mass (Ws) was achieved. Samples were then transferred to a desiccator with saturated potassium sulfate solution. Weights were recorded every 24 h until equilibrium was reached (fully hydrated mass, Wh). Water absorption capacity was calculated using Equation (5).

(5) $$\%\mathrm{Absorption}\;\mathrm{Capacity}=\frac{W_{h}-W_{s}}{W_{s}}\times 100\%$$

#### 2.5.8. Contact Angle in Water and Oil

A film sample was placed on a horizontal white background surface. A 5 µL droplet of distilled water (colored with a trace amount of dye for visualization) or soybean oil was carefully deposited onto the film surface using a calibrated micropipette. Images were captured 30 s after deposition using a digital camera positioned at a fixed distance of 20 cm from the sample. The contact angle was determined using Goniotrans software (version 1.4.0, developed by Felipe Gordillo, Elche, Spain) by analyzing the droplet profile (sessile drop method). Measurements were performed in triplicate for each formulation, and results were expressed as mean ± standard deviation [18].

#### 2.5.9. Cumulative Weight Loss and Gain

The methodology of Gómez-Contreras et al. [19] was followed with adjustments. Films were cut into 2 × 2 cm pieces and placed in a desiccator with calcium chloride (0% RH) at room temperature. Weights were recorded every 24 h until constant (72 h), considered the dry weight. Films were then transferred to a desiccator with saturated potassium sulfate solution and weighed every 24 h until nearly constant (72 h), considered the fully hydrated weight. Graphs were constructed showing cumulative weight loss or gain for each formulation, with each value representing the accumulated change per time interval (see Equations (6) and (7)). The procedure was performed in triplicate.

(6) $$\mathrm{Cumulative}\;\mathrm{weight}\;\mathrm{loss}=P_{0}-P_{t}$$

(7) $$\mathrm{Cumulative}\;\mathrm{weight}\;\mathrm{gain}=P_{t}-P_{0}$$

where P_0_ is the initial film weight and P_t_ is the film weight at time t

#### 2.5.10. Mechanical Properties

Mechanical properties were determined according to Acevedo-Puello et al. [13]. Elastic modulus (EM), tensile strength (TS), and elongation at break (E) were measured using a texture analyzer (TX-700 Texture Analizer, (LAMY Rheology, Champagne-au-Mont-d’Or, France) with a 500 N load cell operating at 50 mm/min crosshead speed. Film samples measured 1 cm × 100 cm with a 5 cm grip distance.

#### 2.5.11. Microstructure

To analyze surface structural properties, 1 × 1 cm film samples were observed under an optical microscope (ZEISS model 415500-1800-000, Carl Zeiss AG, Oberkochen, Germany) at 10× magnification. Films were previously conditioned at 52.8% RH and 25 °C for one week [20]. Cross-sectional microstructure was examined using a scanning electron microscope (JSM-5910, JEOL Ltd., Tokyo, Japan). Samples were dried in desiccators with P_2_O_5_ for 2 weeks to ensure complete dehydration, cut into 0.5 cm pieces, mounted on copper stubs, gold-coated, and observed at 10 kV accelerating voltage [21].

#### 2.5.12. Evaluation of Film Performance in a Food Matrix

All film formulations based on poly(vinyl alcohol) (PVA) and *Clitoria ternatea* L. extract were exposed to a real food matrix to assess their colorimetric response during spoilage. Fragments of each formulation, previously labeled and individually suspended using threads to avoid direct contact, were placed inside a zip-type polyethylene bag. A fresh shrimp was introduced into the system. The bag was hermetically sealed and stored at 5 °C for 12 days under controlled refrigerated conditions. This setup allowed shrimp-released vapors to interact with the films without direct contact, enabling observation of color changes during storage.

#### 2.5.13. pH and Titratable Acidity

pH was determined using a digital pH meter equipped with a glass–calomel electrode. A homogenized shrimp sample (10 g) was mixed with 100 mL of distilled water (1:10 *w*/*v*) to obtain a pulp suspension. The electrode was directly immersed in the homogenate for measurement [22].

Titratable acidity was measured according to Estefanía et al. [23]. A 10–20 g homogenized sample was weighed into a beaker, mixed with 100 mL of distilled water, and allowed to stand briefly before titration. The mixture was titrated with 0.1 N NaOH using phenolphthalein as a visual indicator until a pale pink endpoint was reached. Acidity was calculated from the volume of NaOH consumed using Equation (8).

(8) $$\%Acidez=\frac{N\times V1\times Meq}{m}$$

where

N = sodium hydroxide concentration (normality).
V_1_ = volume of sodium hydroxide consumed (mL).
mEq = milliequivalent of the predominant acid in the sample.
m = sample mass (g).

#### 2.5.14. Color Determination

Color was measured with a colorimeter, obtaining CIE Lab* coordinates. In this system, L* represents vertical luminosity (0 = black, 100 = white), while a* and b* indicate horizontal orientation toward red–green and yellow–blue, respectively [24]. Total color change (ΔE*) was calculated by comparing shrimp pulp coordinates to a reference sample using Equation (9).

(9) $$\Delta L*={L*}_{\mathrm{sample}}\;-\;{L*}_{re}𝒻\;\;\Delta b*={b*}_{\mathrm{sample}}\;-\;{b*}_{re}𝒻\;\;\Delta a*={a*}_{\mathrm{sample}}\;-\;{a*}_{re}𝒻\;\Delta E\;*=\sqrt{{∆L\;*}^{2}+{∆b\;*}^{2}+{∆a\;*}^{2}}$$

where:

L*_sample_, a*_sample_, b*_sample_ = color parameters of the sample
L*_re_𝒻, a*_re_𝒻, b*_re_𝒻 = color parameters of the reference

#### 2.5.15. Total Volatile Basic Nitrogen (TVB-N)

TVB-N quantification followed the method in [25]. Ten grams of minced shrimp tissue were homogenized with 90 mL 6% perchloric acid for 1 min. The mixture was centrifuged at 3000 rpm for 5 min, and the supernatant was filtered. Fifty mL of filtrate were alkalized (checked with phenolphthalein), transferred to a distillation unit with 10 mL 20% (*w*/*v*) NaOH. The distillate was collected in 100 mL 3% (*w*/*v*) boric acid. A blank was run simultaneously using 50 mL 6% perchloric acid instead of extract. The solution was titrated with 0.01 N HCl using Shiro T-Shiro indicator. TVB-N content was calculated using Equation (10), where V_1_ is HCl volume for sample titration (mL), V_2_ is HCl volume for blank (mL), and m is sample mass (g).

(10) $$\frac{mg\;(N)}{100\;g\;sample}=\frac{(V1-V2)\times 0.14\times 2\times 100}{m}$$

where:

V_1_ = volume of hydrochloric acid consumed during sample titration (mL).
V_2_ = volume of hydrochloric acid consumed during blank titration (mL).
m = sample mass (g).

#### 2.5.16. First-Order Kinetic Model for Shrimp Spoilage Evaluation via TVB-N Formation

To monitor chemical spoilage of shrimp during storage, a first-order kinetic model based on TVB-N formation was applied. This internationally recognized indicator for fishery products quantifies accumulation of nitrogenous compounds from proteolytic decomposition by microbial and enzymatic action. Progressive increase in these bases accurately reflects temporal loss of freshness. Fresh shrimp were purchased locally, transported under cold chain conditions, classified, and stored in uniform batches at 5 °C throughout the study. The first-order kinetic model describing deterioration behavior is represented by Equation (11):

Q = Q_0_ ± kt (11)

where:

Q_0_ = initial quality attribute value
Q = final value after time t
k = rate constant, t = elapsed time.

### 2.6. Statistical Analysis

Statistical evaluation was performed using analysis of variance (ANOVA). Significant differences (*p* < 0.05) were assessed with Fisher’s least significant difference test across analyses of different samples. Statistical computations were carried out using Statgraphics Centurion XVI software (version 16.1.11, Manugistics Corp., Rockville, MD, USA).

## 3. Results

### 3.1. Extract Characterization

#### 3.1.1. Anthocyanin Quantification

Regarding the anthocyanin quantification obtained in the assays, the solid-to-liquid ratio used in the extraction can be observed in Table 2. A high anthocyanin content was obtained from the Asian pigeonwings flower (*Clitoria ternatea* L.). This value can be compared with that reported by Handayani et al. [10], who reported a similar total anthocyanin content (125.24 ± 1.29 mg/L) under one of their extraction conditions, although they obtained markedly higher values (up to 551.06 ± 1.01 mg/L) with other solvent combinations. It can be affirmed that an optimized treatment yields a greater anthocyanin content. It is also evident that increasing the water content and decreasing the HCl concentration in the extraction solvent reduces the extracted anthocyanin content, while increasing the ethanol content improves it, since ethanol proves more effective for anthocyanin extraction. The anthocyanins in this flower encompass hydrophilic components as well as water-insoluble lipophilic compounds such as fatty acids, phytosterols, and tocols [26].

The anthocyanin concentration obtained in this study was comparable to that reported by Chandra Singh et al. [27]. Although the authors recognize that raw material variety can be a major source of variation in anthocyanin profile and content, in this study the variability is particularly relevant due to the use of the extract as a colorant in a film developed for freshness monitoring. Additionally, some observed differences may be related to inherent limitations of each analytical method used for measurement, which also influence the sensitivity and color stability of the indicator materials.

#### 3.1.2. UV-Vis Characterization

**Figure 1:**
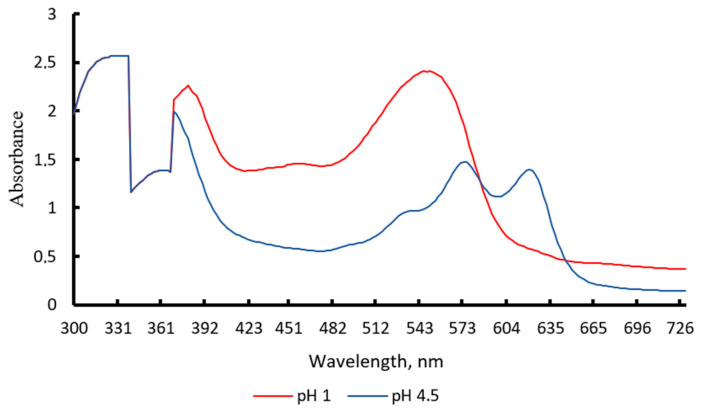
UV–Visible spectra of anthocyanins in pH 1.0 and 4.5 buffers.

**Figure 2:**
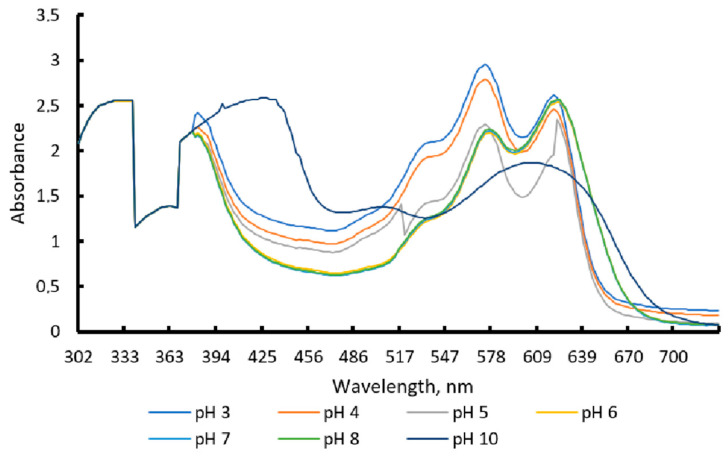
UV–Vis spectra of the anthocyanin extract from blue pea flower (*Clitoria ternatea* L.) at different pH values.

In Figure 1, it is observed that, in the pH-differential spectrophotometric method, the anthocyanin pigments do not exhibit zero absorbance at pH 4.5. This is consistent with the fact that a fraction of the pigment remains in partially colored forms, generating residual absorbance. Anthocyanins exhibit reversible color changes as a function of pH (Figure 2). This characteristic may limit their stability when used as traditional food colorants; however, it is highly advantageous for the development of intelligent films or freshness indicators. Thanks to these color transitions, it is possible to monitor pH variations associated with food degradation and, at the same time, use this behavior to easily estimate the total pigment concentration present in the material [11].

**Figure 3:**
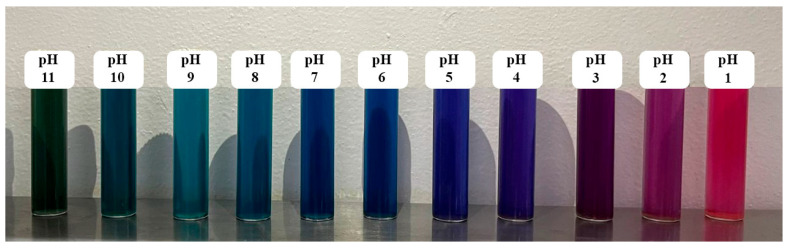
Extract behavior at different pH values.

Figure 2 shows the UV-Vis spectrum of the evaluated anthocyanin extract at different pH levels. A clear shift in the maximum absorption peak is observed within the visible region, indicating that both the maximum wavelength (λmax) and maximum absorbance vary with pH. This behavior confirms the high sensitivity of the pigment to changes in the medium. In acidic conditions (pH 3 and 4), the extract exhibits λmax values of 574 nm and 570 nm, respectively, associated with pink and violet tones (see Table 3 and Figure 3). At intermediate pH (5 and 6), the absorption maximum shifts to shorter wavelengths (320–338 nm), where blue color predominates.

At near-neutral and slightly basic values (pH 7 and 8), the spectrum shifts toward wavelengths in the visible region (662 and 622 nm), corresponding to lavender and light blue colors. This progressive change confirms the pH-dependent nature of anthocyanins, a fundamental characteristic for their application in intelligent films for freshness monitoring (Figure 2).

### 3.2. Film Characterization

#### 3.2.1. Optical and Color Properties

The optical and color properties of the films prepared from PVA with *Clitoria ternatea* L. flower anthocyanin extracts are presented in Table 4. Gloss at 60°, CIE Lab* parameters, hue angle, chroma, and color difference were determined, providing a detailed view of the visual appearance of the obtained materials.

The optical properties of the studied formulations vary according to the addition of anthocyanin extract (EAC). The films progressively darkened, as evidenced by the decrease in luminosity (L*), and exhibited more intense tones toward green and blue, indicated by negative a* and b* values. Furthermore, increasing anthocyanin content generated more saturated colors and more perceptible color changes relative to the control, observed in higher chroma (C) and total color difference (ΔE) values. Gloss also followed a similar trend, increasing from 18 (PA-C) to 41 (PA-A30). This can be attributed to anthocyanins, which are highly sensitive to the chemical conditions of the medium and possess a molecular structure that interacts intensely with light. Additionally, their flavonoid structure contains conjugated double bonds that enable absorption of visible light, particularly at wavelengths corresponding to red, purple, and blue colors [28]. As anthocyanin content in the film increases, greater light absorption occurs, manifesting as reduced luminosity (L*) and the appearance of darker colors. Simultaneously, the specific chemical form adopted by the pigment within the polymeric matrix—determined by factors such as pH and molecular interactions—promotes structures that reflect bluish and greenish tones [29]. This explains the negative values recorded in the a* and b* coordinates. From an optical perspective, higher pigment concentration intensifies selective light scattering and absorption phenomena, altering both gloss and color purity (C). Consequently, the progressive increase in ΔE demonstrates the significant chromatic effect exerted by anthocyanin incorporation into the material.

In summary, anthocyanin incorporation significantly modifies the visual appearance of the films, imparting darker and bluer tones as their proportion in the formulation increases.

#### 3.2.2. Transmittance and Opacity

**Figure 4:**
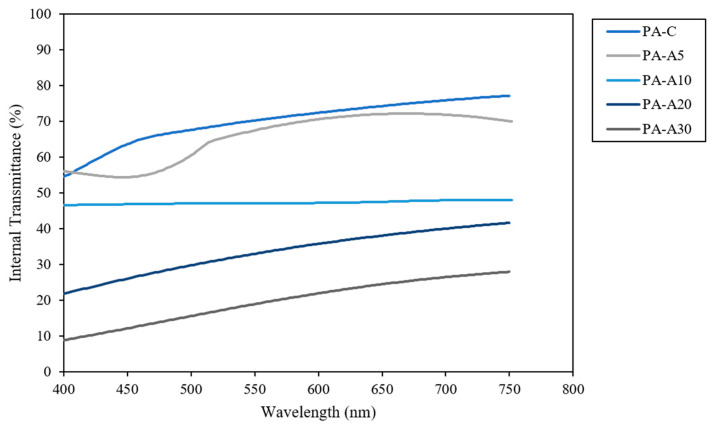
Direct UV-Vis transmittance spectra of the different treatments.

Figure 4 shows the direct transmittance spectra in the UV-Vis range (200–900 nm) of the films prepared from PVA with EAC addition. Transmittance measures the passage of light through the films [30], thereby indicating the transparency of the studied films.

The control film (PA-C) exhibited the highest transmittance across the visible spectrum, remaining around 47–48%, demonstrating high transparency and low light absorption. With the addition of small amounts of anthocyanin (PA-A5 and PA-A10), transmittance decreased moderately, especially in the 400–500 nm region corresponding to the maximum absorption of these pigments. This reduction was more pronounced in PA-A10 than in PA-A5, confirming greater light absorption with increasing colorant concentration. At higher concentrations (PA-A20 and PA-A30), the decrease in transmittance was even more marked. PA-A20 recorded values between 25% and 42%, while PA-A30 showed the lowest transmittance (10–28%), indicating a notably darker and less transparent material. This behavior is consistent with the strong capacity of anthocyanins to absorb light in the visible region, particularly at short wavelengths (400–500 nm), explaining the greater opacity observed in films with higher pigment loading [31].

In Table 5, the opacity values of the films prepared from PVA with EAC are presented. Opacity relates to the fraction of light blocked or absorbed by the film and is inversely related to transmittance. In this sense, the control film (PA-C), formulated without anthocyanin, recorded the lowest opacity value (1.27), confirming its high transparency compared to the other samples. The incorporation of increasing amounts of anthocyanin (PA-A5 and PA-A10) generated a progressive increase in opacity, attributable to the pigment’s capacity to absorb radiation in the UV-Vis range and thereby reduce light passage through the material. This trend aligns with the previously reported transmittance patterns.

In formulations with higher pigment concentration (PA-A20 and PA-A30), opacity increased substantially, reaching its maximum value in PA-A30 (4.52). This result reflects a notably darker film with lower light transmission, evidencing the opacifying effect of anthocyanin. This behavior is explained by the highly conjugated molecular structure of the pigment, which favors intense absorption of visible light [32]. Statistical differences among samples (indicated by different superscripts, *p* < 0.05) validate that each increase in anthocyanin concentration generates a significant change in opacity, ruling out random variation.

These findings confirm anthocyanin’s role as a light-absorbing agent, with efficacy intensifying proportionally with its concentration. This direct relationship is explained by the conjugated molecular structure of the pigment, which enhances absorption of ultraviolet and visible radiation, imparting protective properties against light exposure to the material.

#### 3.2.3. Physical Properties and Water Absorption

Table 6 presents the mean values and standard deviation of the physical properties and water absorption of the films developed from PVA with EAC. The evaluated parameters include thickness (μm), water vapor permeability (WVP), moisture content (Xw), and water absorption capacity (WCA) of the studied films. Incorporation of EAC modified the physical properties of the obtained materials relative to the control film.

Thickness increased with EAC incorporation, reaching maximum values in PA-A20. This increase can be attributed to the additional solid content introduced by the extract and to the establishment of intermolecular hydrogen bonding between hydroxyl groups of PVA and phenolic groups present in the anthocyanins. These interactions likely promote partial molecular rearrangement and increased matrix compactness during film formation, contributing to changes in thickness. The extract is expected to remain physically entrapped within the polymeric network rather than forming covalent bonds. Regarding water vapor permeability (WVP), an initial increase was observed in formulation PA-A5, followed by a reduction at higher concentrations. At low extract levels, the incorporation of anthocyanins may disrupt the continuity of the polymer network, increasing free volume and facilitating water vapor diffusion. At higher concentrations, however, enhanced intermolecular interactions and possible matrix reorganization may reduce molecular mobility and improve barrier properties [33], who analyzed PVA films with purple tomato anthocyanin extract and found that thickness increases with higher extract concentration, while WVP also varies. On the other hand, water absorption capacity (WCA) decreased significantly in all formulations containing anthocyanins compared to the control (PA-C), reflecting lower hydrophilicity. This is likely due to interactions between PVA hydroxyl groups and phenolic compounds in the extract, reducing the availability of hydrophilic sites for water binding [34]. Moisture content (Xw) showed similar values across all formulations, indicating that extract addition does not significantly alter residual moisture in the dry material. Nevertheless, the integrated analysis of thickness, water vapor permeability, and absorption capacity reveals that anthocyanins induce structural modifications that directly affect the stability and functional performance of the films [35].

Therefore, although moisture content (Xw) showed no significant differences, the combined relationship of increased thickness, reduced water absorption, and variations in permeability demonstrates that anthocyanins act as structuring agents within the PVA matrix, altering molecular organization and, consequently, functional performance as a packaging material.

#### 3.2.4. Contact Angle

**Figure 5:**
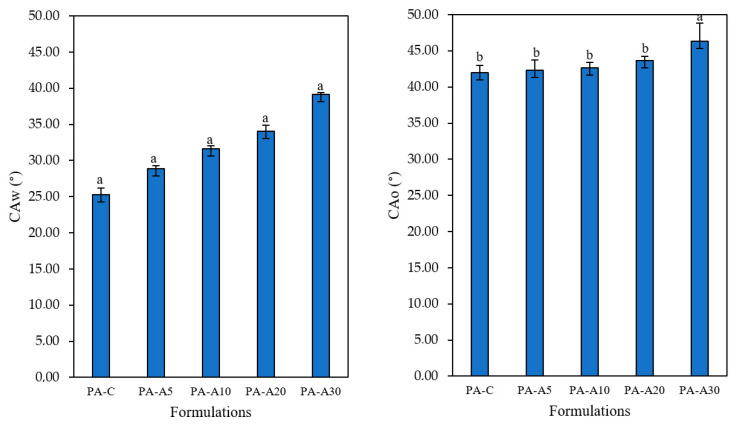
Mean values and standard deviation of water contact angle (CAw, °) and oil contact angle (CAo, °) of the studied films. Different superscript letters indicate statistically significant differences (*p* < 0.05) among formulations.

Figure 5 compares the water contact angle (CAw) and oil contact angle (CAo) for different film formulations developed from PVA with EAC addition. The contact angle is an indicator of the hydrophilic or hydrophobic properties of the films. Addition of anthocyanin extract (EAC) produced a significant modification in the water contact angle, which increased progressively from 25.3° in the control film (PA-C) to 39.13° in formulation PA-A30. This increase evidences a reduction in surface hydrophilicity as extract concentration rises, a trend that correlates with the previously observed decrease in water absorption capacity (WCA). The phenomenon can be attributed to interactions between phenolic compounds in the extract and PVA hydroxyl groups, reducing the availability of active sites for water molecule binding [34]. This explanation consistently integrates both reduced surface hydrophilicity and lower water absorption. Collectively, the results support the hypothesis that anthocyanins exert a structuring effect in the polymeric matrix, generating a surface less affinitive to water and, consequently, with greater potential stability in humid environments. In contrast, the oil contact angle (CAo) showed relatively homogeneous values among formulations, with no significant differences between the control film (PA-C) and those with low or moderate extract concentrations. However, formulation PA-A30 exhibited a considerable increase compared to the other groups. This behavior indicates that material oleophobicity remains practically unaltered with reduced or intermediate anthocyanin incorporation but can increase significantly at higher concentrations [36].

The behavior toward water and oil demonstrates that the extract selectively modifies surface affinity, predominantly affecting interaction with polar substances. Collectively, the contact angle, water absorption, and permeability data show structural coherence and confirm that extract incorporation not only alters internal film morphology but also modulates surface behavior toward liquids of different polarities.

#### 3.2.5. Mechanical Properties

Mechanical properties allow assessment of film strength and flexibility. Table 7 shows the mean values and standard deviation of mechanical properties (EM: elastic modulus, TS: tensile strength, E: elongation at break) of the studied formulations.

TS values refer to the maximum resistance of the films before rupture. A decrease in this parameter is observed as (EAC) increases. Similarly, EM values, a key indicator of material rigidity, exhibit the same behavior. Additionally, E values show a direct proportional relationship with (EAC). This may be related to interactions between the extract and the polymeric structure. Its phenolic compounds, containing multiple hydroxyl groups, interfere with hydrogen bonds that maintain the polymeric network. This reduces internal cohesion, increases chain mobility, and produces a plasticizing effect. Furthermore, extract addition may create microscopic irregularities and decrease polymer crystallinity, preventing ordered chain organization. As a result, the matrix becomes less rigid and more flexible, reducing tensile strength [37]. Similar behavior was reported by [38], where biodegradable chitosan and poly (vinyl alcohol) (PVA) films with *Clitoria ternatea* L. anthocyanin extract showed decreased TS and EM values with increasing extract, achieving tensile strength of 11.02 MPa and elongation of 48.00%. According to one study, optimal tensile strength in edible films is 11.417 N/mm^2^. However, mechanical stability may decrease with excessive ingredient addition, while anthocyanin incorporation contributes to increased film flexibility [39]. According to research published in [39], the incorporation of anthocyanins from red cabbage into κ-carrageenan/CMC films induced notable alterations in their mechanical properties. Specifically, a slight increase in tensile strength (TS) was observed at low dosages of the extract, which is attributed to the formation of hydrogen bonds between the pigments and the polymer matrix, resulting in a denser network. However, upon increasing the pigment concentration, a decrease in TS and a more pronounced reduction in elongation at break (EAB) were recorded, reflecting a loss of flexibility and an increase in material rigidity. These findings suggest that while anthocyanins can act as reinforcing agents at low concentrations, an excess of them disrupts the cohesion of the polymeric structure. Consequently, when contrasting these results with those of other intelligent films, the existence of an optimal anthocyanin level that *harmonises* acceptable mechanical performance with its capacity to respond as an indicator is confirmed.

The molecular weight of Polyvinyl Alcohol (200,000 g/mol) is a key factor in the mechanical properties of the system, as longer polymer chains promote greater entanglement and a higher density of hydrogen bonding interactions, contributing to an initially more resistant and cohesive matrix. However, the progressive incorporation of the additive alters these intermolecular interactions, reducing the entanglement efficiency characteristic of a high molecular weight PVA [40]. Consequently, a decrease in tensile strength and elastic modulus is observed, along with an increase in elongation at break, indicating a more ductile behaviour associated with greater mobility of the polymer chains.

#### 3.2.6. Microstructure

**Figure 6:**
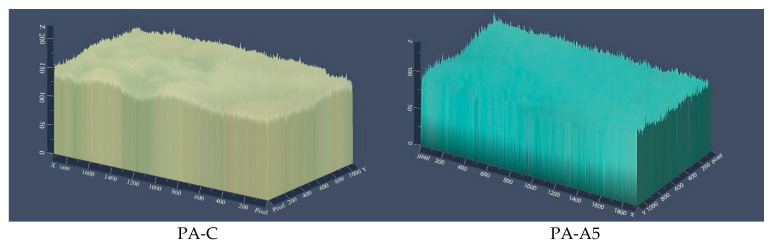

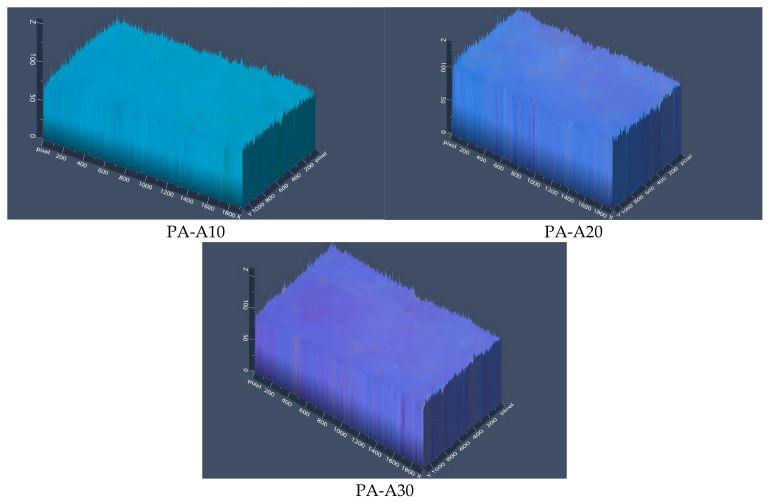
2.5D surface optical micrographs (10×) of the studied biodegradable films.

In Figure 6, 2.5D optical micrographs of the studied films are presented, allowing qualitative observation of surface roughness and elevation. All samples exhibit structurally intact morphology without apparent discontinuities, indicating adequate polymeric matrix formation. The reference film (PVA-C) shows relatively uniform topography, characteristic of the unmodified polymer. With progressive anthocyanin incorporation, slight variations in surface texture can be observed, particularly in formulations PVA-A20 and PVA-A30. Although these differences are not markedly pronounced, subtle increases in surface irregularity may be associated with the presence and dispersion of extract components within the polymeric matrix, as well as possible intermolecular interactions between phenolic hydroxyl groups and PVA chains, which can promote localized heterogeneity [41].

Such textural variations, even when moderate, may influence functional properties such as permeability, wettability, and colorimetric responsiveness. Previous studies have reported that incorporation of polyphenolic compounds into biopolymer matrices can induce surface irregularities and microheterogeneous domains, supporting this qualitative observation [42].

#### 3.2.7. Evaluation of Film Performance in a Food Matrix

The shrimp was selected as a study model because it is a highly perishable marine product that undergoes rapid biochemical and microbiological changes after harvest. During spoilage, shrimp exhibit appreciable increases in pH and generate volatile nitrogenous compounds, such as ammonia and trimethylamine, widely recognised as indicators of freshness loss. These characteristics make it a suitable matrix for evaluating the responsiveness of colorimetric indicators intended for applications in intelligent packaging. However, since the detection mechanism of the films is based on interaction with basic volatile compounds associated with protein degradation, their potential application is not limited exclusively to shrimp but could extend to other protein-rich food matrices with similar spoilage patterns, such as fish, other seafood, and fresh meats. Nevertheless, specific studies would be necessary to validate their performance in each particular system.

**Figure 7:**
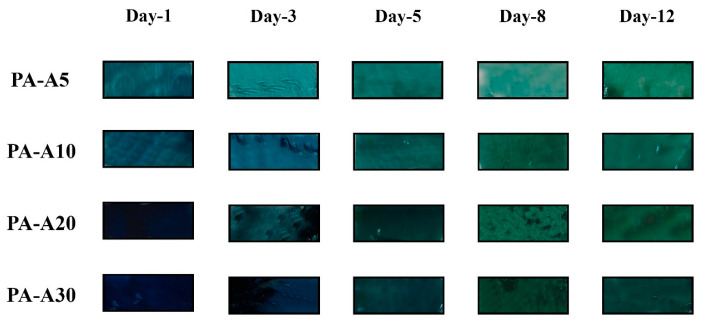
Color changes of PA-A5, PA-A10, PA-A20, and PA-A30 films in the presence of volatile ammonia vapor.

Color responses of each film at different pH values are presented in Table 8. Films prepared with *Clitoria ternatea* L. extract showed clearly time- and anthocyanin concentration-dependent color variations (see Figure 7). On day 0, all formulations exhibited light or dark bluish tones. During days 3, 5, 8, and 12, due to gradual interaction with volatile compounds released by shrimp (stored with the films in hermetic Ziploc^®®^-type bags), a progressive transition to greenish-blue or green tones was observed, especially in formulations with higher extract concentration (PA-A20 and PA-A30). This is evidenced by increased b* (tendency toward yellow) and decreased a* (greater tendency toward green), as well as changes in hue angle h, which increased in several formulations as storage progressed. Luminosity values (L*) also varied over time, showing initial increases followed by stabilization, coinciding with slight surface lightning during the first days. Chroma (c*) decreased more markedly in formulations with lower anthocyanin concentrations, suggesting gradual loss of color saturation as films reacted with volatile nitrogenous compounds [43,44].

Although the present study demonstrates the effectiveness of the films as non-contact colourimetric indicators, the potential migration of anthocyanins from the PVA matrix into food systems represents an important safety consideration. Due to the hydrophilic nature of both poly(vinyl alcohol) and anthocyanins from *Clitoria ternatea*, diffusion of bioactive compounds could occur under conditions of direct contact, high moisture content, or prolonged storage. In the experimental design employed here, film fragments were suspended inside a sealed system without direct contact with the shrimp, thereby limiting migration through physical transfer or surface moisture interaction. Nevertheless, this configuration does not replace a standardised migration assessment. Therefore, future investigations should include global and specific migration tests using appropriate food simulants, followed by spectrophotometric or chromatographic quantification of released anthocyanins. Such studies would allow a more comprehensive evaluation of the material’s compliance with food-contact safety regulations and contribute to the optimisation of film formulation for commercial intelligent packaging applications.

Total color difference (ΔE) showed perceptible variations in all formulations, with values well above the threshold ΔE > 5, indicating clearly visible differences to the naked eye. PA-A20 and PA-A30 exhibited the largest changes between days, reaching ΔE values above 20 at certain points, confirming their sensitivity to chemical environmental changes caused by shrimp spoilage [45].

Overall, these results demonstrate that the films respond differently depending on incorporated anthocyanin concentration and confirm their capacity to detect volatile compounds associated with spoilage, exhibiting color change transitions that can be interpreted visually without specialized instruments. As stated by Zhao et al. [46], these films have potential for use as colorimetric sensor labels. However, to date, the use of natural pigments for manufacturing pH-sensitive intelligent colorimetric products has not been widely reported. Additionally, film RGB values can be converted to total volatile basic nitrogen (TVB-N) values and analyzed automatically via a smartphone application to determine meat freshness [47].

**Figure 8:**
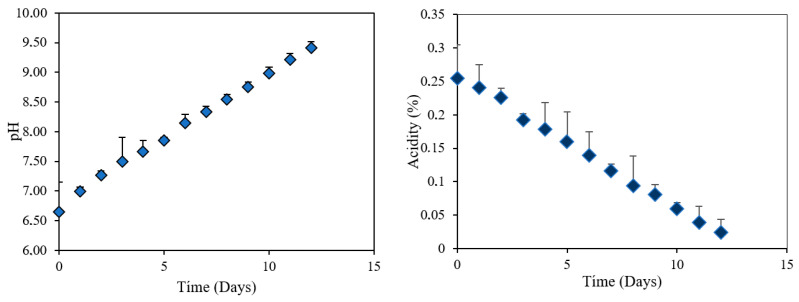
Variation in pH (**left**) and titratable acidity (**right**) in shrimp exposed to intelligent films with anthocyanin extract during storage at 5 °C.

Although total color difference (ΔE) values exceeded 5 in all formulations—indicating clearly perceptible color changes to the naked eye and thereby confirming the films’ sensitivity to chemical changes induced by shrimp spoilage a limitation inherent to the anthocyanins from *Clitoria ternatea* must be acknowledged. In the typical pH range of shrimp muscle during storage and spoilage, these anthocyanins maintain a dominant blue coloration with only subtle variations in intensity, transitioning from light blue to bluish-green or greenish-blue, rather than exhibiting sharp or dramatic color shifts. As evidenced in Figure 8 and Table 3, these changes are reliably quantifiable using instrumental methods; however, visual perceptibility may be less pronounced under practical conditions, such as variable lighting or in the absence of direct side-by-side comparison, potentially limiting the indicator’s utility for rapid, unaided visual assessment by non-expert users [48].

To overcome this limitation and enhance visual contrast in the alkaline pH ranges relevant to seafood spoilage, future formulations could explore blending *C. ternatea* anthocyanins with other natural pigments known for more pronounced pH-dependent transitions, such as anthocyanins from red cabbage or curcumin. Such hybrid systems may produce larger ΔE values and clearer visual cues while retaining the advantages of natural and sustainable indicators [49].

These results can be compared with those of [9], who state that this color behavior relative to the film and food matrix may reflect pH changes during the decomposition process.

#### 3.2.8. pH and Titratable Acidity in the Studied Shrimp

In Figure 8, during refrigerated storage at 5 °C, shrimp pH showed a continuous increase from an initial value of 6.6 to approximately 9.2 at the end of the period. This progressive alkalinization is characteristic of spoilage in fishery products, attributed to accumulation of volatile nitrogenous compounds such as ammonia, trimethylamine, and other amines generated by microbial and enzymatic activity [50]. Therefore, the upward pH trend reflects gradual loss of initial freshness. Conversely, titratable acidity decreased progressively from 0.25% to 0.03%. This reduction is explained by microbial metabolic consumption of organic acids, which simultaneously generate basic metabolites. The antagonistic relationship observed between pH and acidity is consistent with typical biochemical patterns of decomposition in hydrobiological resources [41]. These results validate the suitability of the intelligent anthocyanin film as a freshness indicator system, given that its color responds to pH variations. As spoilage advances and the medium becomes alkaline, the film undergoes a visible chromatic transition that allows evaluation of product status without instrumental techniques. Thus, the systematic correlation among pH increase, acidity decrease, and material visual response confirms its potential as a quality monitoring tool in refrigerated marine products.

#### 3.2.9. Total Volatile Basic Nitrogen (TVB-N)

**Figure 9:**
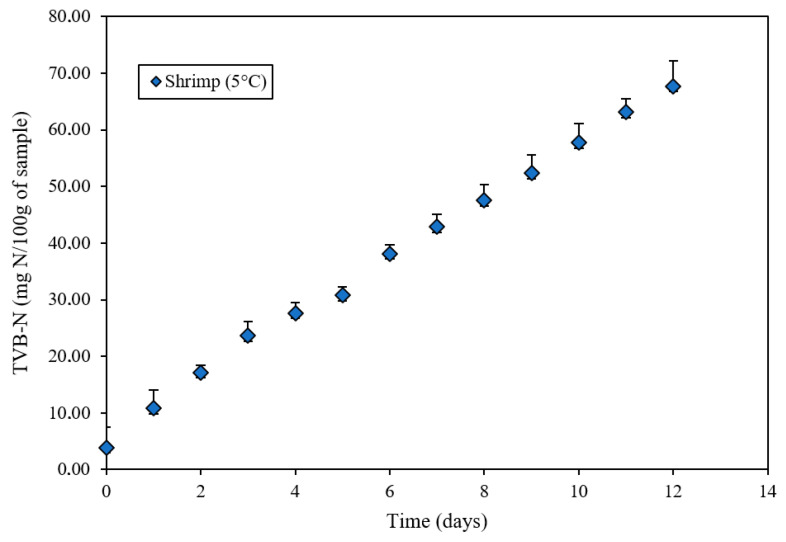
TVB-N formation in shrimp in contact with an intelligent film formulated with anthocyanin extract during storage at 5 °C.

In Figure 9, the progressive formation of total volatile basic nitrogen (TVB-N) during storage is observed, showing a clear correlation with the parallel increase in pH of the shrimp samples. This relationship is explained by the chemical nature of TVB-N components—ammonia, trimethylamine, and dimethylamine—alkaline compounds generated through microbial and enzymatic degradation of nitrogenous constituents in shrimp muscle [50]. The gradual accumulation of these volatile bases led to a systematic increase in alkalinity, reflected by the rise in pH from initial values of approximately 6.5 to levels exceeding 9.0 at the end of storage.

Progressive release of these volatile bases generates a systematic increase in medium alkalinity, manifested in the pH rise from initial values of 6.5 to levels above 9.0 at the end of storage. The close correspondence between these two variables evidence that both constitute complementary indicators of the spoilage process, simultaneously validating that the colorimetric response of the intelligent film is directly associated with accumulation of characteristic alkaline volatile compounds of refrigerated shrimp degradation [51].

#### 3.2.10. First-Order Kinetic Model for Evaluating Shrimp Spoilage via TVB-N Formation

**Figure 10:**
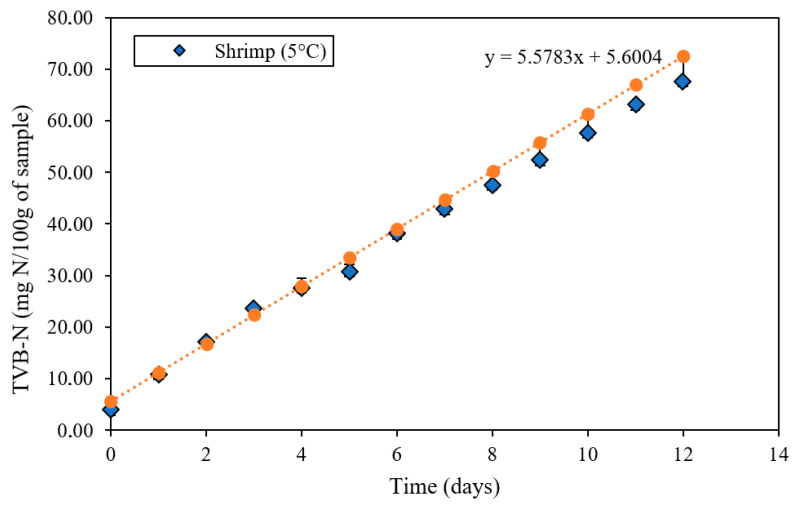
First-order kinetic linear regression of total volatile basic nitrogen (TVB-N) formation in shrimp in contact with an intelligent film formulated with anthocyanin extract during storage at 5 °C.

Figure 10 and Table 9 show the kinetics of total volatile basic nitrogen (TVB-N) formation and the kinetic parameters of the first-order model in refrigerated shrimp at 5 °C covered with intelligent anthocyanin films. The temporal evolution of TVB-N exhibits a linear relationship with first-order kinetic fit, demonstrating a direct correlation between storage time and accumulation of these compounds. This sustained increase reflects typical proteolytic decomposition and microbial development processes in refrigerated fishery products. The slope of the kinetic equation (5.5783) quantifies the generation rate of basic volatile compounds, predominantly ammonia, trimethylamine (TMA), and dimethylamine (DMA), originating from degradation of proteins and nitrogenous precursors such as trimethylamine oxide [52]. Progressive accumulation of these substances constitutes a reliable indicator of freshness and sensory quality loss in crustaceans, justifying its inclusion in international quality regulations [53].

Regarding growth parameters (see Table 9), these reveal a high-rate constant (k = 5.57 day^−1^), confirming rapid generation of volatile compounds despite refrigeration, characteristic of shrimp high perishability. Initial TVB-N content (Q_0_ = 5.60 mg/100 g) and estimated shelf life (4.4 ± 0.23 days) establish the period until reaching the regulatory spoilage limit (generally 30 mg/100 g in European standards). Half-life (t_1/2_ = 2.01 ± 0.15 days) evidences the rapid doubling of initial TVB-N, while the coefficient of determination (R^2^ = 0.997) validates the precision of the kinetic model in describing the spoilage mechanism. These quantitative parameters provide a scientific basis for predicting product behavior during refrigerated storage. Selection of this model was appropriate because it described progressive formation of volatile basic nitrogen (TVB-N) in marine products during refrigerated storage. The study by Wannawisan et al. [52] demonstrated that TVB-N accumulation follows linear behavior with respect to time, showing high coefficients of determination (R^2^ > 0.98), confirming that its increase depends directly on advancement of microbial and enzymatic spoilage.

## 4. Conclusions

The incorporation of anthocyanin extract (EAC) significantly influenced the optical, structural, mechanical, and functional properties of the developed films. Increasing extract concentration intensified the bluish coloration, confirming its ability to absorb UV–visible radiation and contribute to photostability. While moisture content remained statistically unchanged, variations in thickness, water absorption, and permeability suggest that EAC acts as a structuring agent within the PVA matrix. These effects are attributed to hydrogen bonding interactions between anthocyanin hydroxyl groups and the polymer chains, promoting molecular rearrangement, microheterogeneity formation, and increased affinity toward polar compounds. From a mechanical perspective, EAC exhibited a plasticizing effect, reducing tensile strength and elastic modulus while increasing elongation at break. This highlights the importance of optimizing extract concentration to balance flexibility and structural integrity. Functionally, the developed system demonstrated effective performance as an intelligent colorimetric indicator for monitoring refrigerated shrimp freshness. The progressive release of volatile basic nitrogen compounds during spoilage led to pH increases that triggered visible chromatic transitions in the films. The strong correlation among pH, titratable acidity, and total volatile basic nitrogen (TVB-N) confirmed the sensor’s responsiveness to alkaline degradation compounds. Additionally, TVB-N production followed first-order kinetic behavior with a satisfactory statistical fit, supporting the combined use of pH and TVB-N as reliable indicators for shelf-life prediction and intelligent sensor validation. Although the films are composed of poly (vinyl alcohol) (PVA) and natural anthocyanin extracts, materials that have been associated with potential biodegradability in previous studies, the biodegradation behavior of the films developed in this work was not experimentally evaluated. Therefore, future research should address degradation performance under controlled environmental conditions. Furthermore, considering that the sensing mechanism is based on the detection of volatile alkaline compounds derived from protein degradation, the proposed system may also be applicable to other protein-rich food matrices exhibiting similar spoilage pathways, pending specific validation studies.

## Figures and Tables

**Table 1:** Formulation of PVA-based film-forming solutions (2% *w*/*v* PVA, 25% *w*/*w* glycerol) with different levels of volumetric substitution of *Clitoria ternatea* L. extract.

Formulation	PVA	Glycerol	EAC
PA-C	0.8333	0.1667	0.0000
PA-A5	0.8000	0.1600	0.0400
PA-A10	0.7692	0.1538	0.0769
PA-A20	0.7143	0.1429	0.1429
PA-A30	0.6667	0.1333	0.2000

**Table 2:** Total anthocyanin content.

Sample	Solid-to-Liquid Ratio	Total Anthocyanin Content (mg/L)
Ethanol-HCl	1:20	153.15 ± 0.21

The values followed by ± represent the standard deviation of the various extractions.

**Table 3:** Extract behavior at different pH values.

pH	λmax (nm)	Maximum Absorbance	Observed Color
3	574	2.955 ± 0.020 ^a^	Pink
4	570	2.796 ± 0.002 ^b^	Violet
5	320	2.553 ± 0.017 ^c^	Indigo
6	338	2.553 ± 0.009 ^c^	Dark blue
7	662	2.555 ± 0.016 ^c^	Lavender
8	622	2.565 ± 0.030 ^c^	Light blue

Different superscript letters indicate statistically significant differences (*p* < 0.05) among formulations.

**Table 4:** Mean values and standard deviation of gloss (GU) and color parameters (luminosity L*, red/green a*, yellow/blue b*, chromaticity C, hue angle h°) of the studied films.

Formulations	Gloss a 60°	L*	a*	b*	C	h	ΔE	CIE Lab Color
PA-C	18 ± 1.2	88.7 ± 0.33 ^a^	0.78 ± 0.15 ^a^	−0.16 ± 0.15 ^a^	0.813 ± 0.11 ^d^	347.4 ± 1.58 ^a^	-	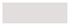
PA-A5	23 ± 1.2	53.4 ± 1.00 ^b^	−11.8 ± 0.30 ^c^	−24.01 ± 0.10 ^c^	26.83 ± 0.81 ^a^	244.6 ± 0.25 ^d^	26.26 ± 4.1 ^d^	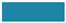
PA-A10	34 ± 1.9	47.9 ± 0.55 ^c^	−19.9 ± 1.92 ^d^	−26.49 ± 1.01 ^d^	22.0 ± 1.98 ^b^	266.7 ± 2.33 ^b^	44.82 ± 3.4 ^c^	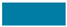
PA-A20	40 ± 4.8	38.0 ± 0.88 ^d^	−11.3 ± 0.85 ^c^	−22.34 ± 1.28 ^b^	2.90 ± 0.87 ^c^	266.3 ± 1.6 ^b^	50.79 ± 1.6 ^b^	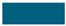
PA-A30	41 ± 6.8	37.1 ± 0.96 ^d^	−2.7 ± 0.51 ^b^	−21.49 ± 1.18 ^b^	2.26 ± 0.72 ^c^	254.8 ± 3.11 ^c^	56.27 ± 1.7 ^a^	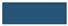

Different superscript letters indicate statistically significant differences (*p* < 0.05) among formulations.

**Table 5:** Mean values and standard deviation of opacity of the studied films.

Formulation	Opacity
PA-C	1.27 ± 0.01 ^d^
PA-A5	1.38 ± 0.08 ^d^
PA-A10	1.90 ± 0.02 ^c^
PA-A20	2.54 ± 0.04 ^b^
PA-A30	4.52 ± 0.03 ^a^

Different superscript letters indicate statistically significant differences (*p* < 0.05) among formulations.

**Table 6:** Mean values and standard deviation of thickness (μm), water vapor permeability (WVP, g-mm/kPa-h-m^2^), water absorption capacity (WCA, g dry film/g wet film) and moisture content (Xw, g water/g dry film) of the studied films.

Formulation	Thickness	WVP	WCA	Xw
PA-C	109.5 ± 0.02 ^bc^	0.77 ± 0.05 ^b^	0.365 ± 0.06 ^a^	0.237 ± 0.07 ^a^
PA-A5	105.3 ± 0.01 ^c^	2.12 ± 0.08 ^a^	0.143 ± 0.06 ^b^	0.177 ± 0.09 ^a^
PA-A10	173.0 ± 0.03 ^ab^	1.79 ± 0.09 ^a^	0.108 ± 0.06 ^b^	0.175 ± 0.01 ^a^
PA-A20	184.1 ± 0.02 ^a^	1.18 ± 0.09 ^a^	0.156 ± 0.01 ^b^	0.197 ± 0.08 ^a^
PA-A30	147.5 ± 0.05 ^a^	1.20 ± 0.08 ^a^	0.133 ± 0.02 ^b^	0.211 ± 0.04 ^a^

Different superscript letters indicate statistically significant differences (*p* < 0.05) among formulations.

**Table 7:** Mean values and standard deviation of mechanical properties (TS: tensile strength, EM: elastic modulus, E: elongation at break) of the studied formulations.

Formulation	TS (MPa)	EM (MPa)	E (%)
PA-C	26.0 ± 2.0 ^a^	91.0 ± 5.0 ^a^	75.2 ± 1.9 ^a^
PA-A5	24.1 ± 1.8 ^ab^	85.0 ± 3.0 ^a^	80.0 ± 2.0 ^b^
PA-A10	22.3 ± 1.4 ^b^	83.0 ± 4.0 ^a^	84.4 ± 1.6 ^c^
PA-A20	19.0 ± 2.0 ^b^	82.0 ± 5.0 ^ab^	86.0 ± 3.0 ^c^
PA-A30	15.2 ± 1.7 ^c^	75.0 ± 3.0 ^b^	92.4 ± 1.5 ^d^

Different superscript letters indicate statistically significant differences (*p* < 0.05) among formulations.

**Table 8:** Mean values and standard deviation of L, a, b, ΔE and color changes of films at different days of exposure with the food matrix.

Days	Parameters	PA-A5	PA-A10	PA-A20	PA-A30
Day 0	L*	30.33 ± 4.04	25.67 ± 4.73	7.00 ± 1.00	5.67 ± 1.15
	a*	−19.33 ± 2.89	−16.33 ± 5.13	1.33 ± 0.58	2.00 ± 1.00
	b*	−14.67 ± 2.52	−17.67 ± 2.89	−11.33 ± 6.35	−17.67 ± 2.52
	c*	24.45 ± 1.25	24.44 ± 2.64	11.51 ± 6.10	17.79 ± 2.56
	h*	232.66 ± 8.64	222.29 ± 12.14	139.87 ± 44.66	145.57 ± 44.66
	ΔE	-	-	-	-
Day 3	L*	42.67 ± 5.13	32.67 ± 3.06	21.00 ± 3.61	15.67 ± 2.08
	a*	−21.33 ± 1.15	−14.67 ± 2.08	−14.00 ± 2.00	−5.33 ± 2.52
	b*	−12.33 ± 6.66	−21.33 ± 1.53	−8.00 ± 1.00	−20.00 ± 1.73
	c*	25.15 ± 2.72	25.97 ± 0.64	16.20 ± 1.23	20.82 ± 1.29
	h*	240.92 ± 13.84	214.51 ± 5.49	239.92 ± 6.63	195.11 ± 6.63
	ΔE	12.40 ± 5.07	9.35 ± 1.17	13.63 ± 2.32	22.50 ± 0.95
Day 5	L*	37.00 ± 7.00	27.00 ± 4.58	13.33 ± 1.53	17.67 ± 1.15
	a*	−28.33 ± 0.58	−25.00 ± 2.65	−12.67 ± 1.53	−16.33 ± 1.15
	b*	−3.33 ± 1.15	−6.00 ± 1.00	−1.33 ± 3.06	−8.67 ± 1.53
	c*	28.54 ± 0.64	25.72 ± 2.63	12.97 ± 1.58	18.55 ± 0.62
	h*	263.31 ± 2.26	256.46 ± 2.36	263.57 ± 13.06	242.01 ± 13.06
	ΔE	15.35 ± 1.47	11.65 ± 1.52	23.64 ± 0.32	15.38 ± 0.54
Day 8	L*	38.33 ± 2.08	27.33 ± 3.21	17.67 ± 2.08	15.33 ± 2.52
	a*	−30.33 ± 0.58	−26.67 ± 2.89	−15.33 ± 5.51	−13.00 ± 4.00
	b*	0.33 ± 2.08	−0.3 ± 0.58	0.33 ± 2.31	3.33 ± 0.58
	c*	30.38 ± 0.55	26.67 ± 2.88	15.43 ± 5.59	13.43 ± 3.99
	h*	270.62 ± 3.96	269.24 ± 1.32	269.54 ± 7.50	284.89 ± 7.50
	ΔE	19.10 ± 2.54	15.78 ± 0.51	21.30 ± 2.21	24.94 ± 2.22
Day 12	L*	40.33 ± 10.26	27.33 ± 2.08	16.67 ± 2.08	14.00 ± 1.00
	a*	−31.67 ± 1.53	−30.33 ± 1.53	−3.67 ± 2.52	−8.33 ± 1.15
	b*	6.33 ± 1.53	5.00 ± 2.65	2.67 ± 2.31	−1.33 ± 1.15
	c*	32.33 ± 1.18	30.80 ± 1.94	4.62 ± 3.23	8.50 ± 1.06
	h*	281.39 ± 3.23	279.20 ± 4.32	296.23 ± 23.41	260.51 ± 23.41
	ΔE	26.73 ± 4.06	22.26 ± 3.26	27.57 ± 0.86	24.54 ± 0.25

**Table 9:** Parameters of the first-order kinetic model for total volatile basic nitrogen (TVB-N) formation in shrimp with biodegradable films.

Temperature (°C)	*k* (day^−1^)	Q_0_ (mg/100 g)	Shelf life (day)	$t_{1/2}$ (day)	$R^{2}$
5 °C	5.57 ± 0.12	5.60 ± 0.10	4.4 ± 0.23	2.01 ± 0.15	0.997

Results are expressed as mean ± standard deviation (SD).

## Data Availability

The data presented in this study are available on request from the corresponding author.
